# Mathematical modeling for the prediction of cerebral white matter lesions based on clinical examination data

**DOI:** 10.1371/journal.pone.0215142

**Published:** 2019-04-16

**Authors:** Yuya Shinkawa, Takashi Yoshida, Yohei Onaka, Makoto Ichinose, Kazuo Ishii

**Affiliations:** 1 Kurume University Graduate School of Medicine, Kurume, Fukuoka, Japan; 2 Shin Takeo Hospital, Takeo, Saga, Japan; 3 Biostatistics Center, Kurume University, Kurume, Fukuoka, Japan; Universidad Miguel Hernandez de Elche, SPAIN

## Abstract

Cerebral white matter lesions are ischemic symptoms caused mainly by microangiopathy; they are diagnosed by MRI because they show up as abnormalities in MRI images. Because patients with white matter lesions do not have any symptoms, MRI often detects the lesions for the first time. Generally, head MRI for the diagnosis and grading of cerebral white matter lesions is performed as an option during medical checkups in Japan. In this study, we develop a mathematical model for the prediction of white matter lesions using data from routine medical evaluations that do not include a head MRI. Linear discriminant analysis, logistic discrimination, Naive Bayes classifier, support vector machine, and random forest were investigated and evaluated by ten-fold cross-validation, using clinical data for 1,904 examinees (988 males and 916 females) from medical checkups that did include the head MRI. The logistic regression model was selected based on a comparison of accuracy and interpretability. The model variables consisted of age, gender, plaque score (PS), LDL, systolic blood pressure (SBP), and administration of antihypertensive medication (odds ratios: 2.99, 1.57, 1.18, 1.06, 1.12, and 1.52, respectively) and showed Areas Under the ROC Curve (AUC) 0.805, the model displayed sensitivity of 72.0%, and specificity 75.1% when the most appropriate cutoff value was used, 0.579 as given by the Youden Index. This model has shown to be useful to identify patients with a high-risk of cerebral white matter lesions, who can then be diagnosed with a head MRI examination in order to prevent dementia, cerebral infarction, and stroke.

## Introduction

In Japan, it is generally recognized that the increase in national health expenditure that accompanies aging is a serious social problem [1]. Although the number of deaths by stroke has decreased drastically due to preventive treatment, stroke still ranks at the top of the health care expenditures in Japan [2]. It is estimated that 2 million people are currently bedridden, and this number will increase to 3 million by 2025 [3]. Therefore, the early detection and prevention of cerebrovascular diseases is important for the reduction of health care expenditures [4].

In recent years, cerebral white matter lesions, which are typically found during MRI tests, are drawing attention as a factor to predict dementia and stroke [5]. It is considered that cerebral white matter lesions are risk factors for dementia and stroke [5], which show progressive symptoms. Cerebral white matter lesions are mainly ischemic symptoms with microangiopathy [6], which show white spots scattered in the cerebral white matter in the MRI image, and gradually progress to a large lump [7].

The surface of the cerebrum is called gray matter, which is composed of gathering nerve cells. There is cerebral white matter at the central side of the gray matter, composed of bundles of nerve fibers that transmit signals from nerve cells. Cerebral white matter is enclosed by cerebral arterioles, which are nutritive vessels. If arteriosclerosis occurs in the cerebral arteriole, the vascular wall loses its elasticity and narrows the vascular lumen, which shows up as white spots on an MRI due to leaked liquids. Because arteriosclerosis is irreversible, if it occurs in the cerebral arterioles, the blood flow to the brain cells chronically decreases, leading to cell necrosis [8].

Most of the patients with cerebral white matter lesions usually engage in work and experience daily life without any problems. For patients without subjective symptoms, the comprehensive medical checkup that includes a head MRI scan, which is called "brain dock," is necessary and is the first choice to detect and diagnose white matter lesions in Japan. Generally, the brain dock is an added option to a comprehensive medical checkup that focuses on brain diseases, and includes diagnostics such as the head MRI. The first brain dock service in Japan was launched in the Shinsapporo Neurosurgical Hospital in March of 1988 [9], after which the brain dock services were expanded to over 600 facilities. As of November 30, 2018, 289 facilities were certified by the facility certification system for brain dock, which is administered by the Japan Brain Dock Society and has been in effect since 2009 [10]. At present, over 670 facilities provide the brain dock course, including those facilities certified by the Japan Brain Dock Society. The brain dock course is a medical evaluation at a patient’s own expense and is not covered by medical insurance. The pricing of brain docks is set independently by each medical facility, and it is said that it generally costs from 50 to 100 thousand yen (approx. 500 to 1000 USD).

According to “Guidelines of brain dock,” by the Japan Brain Dock Society, though head MRI imaging is performed in the brain dock course to detect cerebral white matter lesions and to grade the lesions, because there is no influence and no subjective symptom in the daily life, it is necessary to not to engender unnecessary anxiety. However, for patients with cerebral white matter lesions, it is necessary to pay attention to risk factors and care for adult diseases in order to prevent dementia, cerebral infarction, and stroke from occurring in the future [11].

The goal of this study was to construct a discriminant model to predict cerebral white matter lesions using clinical examination data from a routine medical checkup, toward the development of a screening test based on scientific evidence aiming at the prevention of dementia, cerebral infarction, and stroke.

## Materials and methods

### Subjects

This study is an examination of 1,904 subjects, including 988 males and 916 females, who underwent head MRI and blood tests during the brain dock course of a comprehensive medical checkup sometime between April 1, 2016 and October 31, 2017 at Shin Takeo Hospital. During the investigation period, there were no hospital policy changes for the brain dock course of a comprehensive medical checkup. The average age of the examinees was 56.4 ± 11.5 [range: 20–85] (mean ± S.D.), and 1,044 subjects were diagnosed from their MRI results as having cerebral white matter lesions. In the head MRI, T1 weighted images (T1WI), T2 weighted images (T2WI), and Fluid Attenuated Inversion Recovery (FLAIR) images were obtained by using MRI scanners, MAGNETOM Symphony (Siemens Healthineers Japan, Tokyo, Japan) and MAGNETOM ESSENZA (Siemens Healthineers Japan, Tokyo, Japan).

### Ethical consideration

This study was conducted based on the approval of the ethical review committee of Shin Takeo Hospital. For the protection of patient privacy, the patient data was collected with unlinkable anonymization by a third party, and was saved in a password-protected storage medium for research use only. The clinical data used in this study will be available upon request from readers to the corresponding author based on the data usage agreement and considering the patients’ right of privacy.

## Clinical data

### General inspection and blood and biochemical tests

In the general inspection, six characteristics were recorded for the study: age, gender, systolic blood pressure (SBP), diastolic blood pressure (DBP), body mass index (BMI), and presence of visceral steatosis (for the determination of metabolic syndrome) [12]. The blood and biochemical tests were conducted with laboratory test systems, C8000 (Canon Medical Systems Corporation, Tochigi, Japan) and Acute (Canon Medical Systems Corporation, Tochigi, Japan), respectively. HbA1c was determined with a glycohemoglobin analyzer HA8181 (Arkray inc., Kyoto, Japan). In the blood and biochemical tests, which were conducted as a part of the comprehensive medical checkup, LDL cholesterol (LDL), HDL cholesterol (HDL), LH ratio (quotient of LDL and HDL), Triglyceride (TG), hemoglobin A1c (HbA1c), and blood glucose level (BS) were recorded.

### Ultrasonic testing

The ultrasonic testing was conducted with ultrasonic diagnostic equipments, LOGIQ S7 Expert (GE Healthcare Japan, Tokyo) and Aplio 400 (Canon Medical systems, Tochigi, Japan). Two characteristics were recorded: carotid plaque score (PS) [13] and plaque number (n-plaque). The PS was calculated as follows. The carotid artery was divided into four 15 mm long sections: the central side of the common carotid artery (CCA), the peripheral side of the CCA, the bifurcation of the CCA and the central side of the internal carotid artery. Then, the sum of the maximum values of intima-media thickness exceeding 1.1 mm was calculated.

### Questionnaire in the specific health examination

In the questionnaire in the specific health examination (shown in S1 Doc), six questions were answered by examinees when receiving a comprehensive medical checkup, regarding their experience with medications to reduce blood pressure, medications to reduce blood sugar or insulin injection, medications to decrease the level of cholesterol or of neutral fat, as well as drinking habits (everyday, sometimes, or rarely drink (cannot drink)), drinking volume (less than 180 ml, 180–360 ml, 360–540 ml or more than 540 ml), and smoking habits.

Additional data to replicate all of the figures, graphs, tables, statistics, and other values in this study is available at doi:10.5061/dryad.007467q as Data files: WM_data.

### Assessment of each clinical examination data and questionnaire

R version 3.4.4 and its suitable packages were used to perform all statistical analysis and statistical modeling in this study [14,15]. The relationships between each examination item or each answer of the specific medical examination questionnaire and the probability of the presence of cerebral white matter lesions were investigated. Student's *t* tests [16] were performed for continuous variables and Fisher's exact tests [17] were performed for categorical variables.

### Comparison among various models based on different algorithms

Four kinds of models including linear models, nonlinear models, and a stochastic model were created, and an accuracy comparison among the models was conducted. The normalization of the variables was performed to adjust the factor levels. In the linear modeling, a logistic regression modeling (LogReg) [18] were employed, and in the nonlinear modeling, support vector machine (SVM) [19] or random forest (RF) [20] models were constructed. Furthermore, a Naive Bayes classifior (NB) [21] was constructed as a stochastic model. The probability of the presence or absence of white matter lesions was used as the dependent variable. In each model, the Youden Index [22, 23] was used as the most appropriate cutoff value to compare the performance among models.

The discrete variables (or categorical variables) in this study were treated as follows:

Regarding binary variables of models, 0 or 1 was assigned for absence or presence, respectively, e.g., sex: *X* = 0 for male and *X* = 1 for female; medication to reduce blood pressure: *X* = 0 for “No” and *X* = 1 for “Yes”; medication to reduce blood sugar or insulin injection: *X* = 0 for “No” and *X* = 1 for “Yes”; medication to reduce blood pressure: *X* = 0 for “No” and *X* = 1 for “Yes”.

Regarding ternary variables of models, three kinds of vectors (0,0), (0,1), and (1,1) were assigned for the corresponding combinations with two variables (*X*_1_,*X*_2_), respectively, e.g. the determination of metabolic syndrome: *X*_1_ = 0 and *X*_2_ = 0 for non-metabolic syndrome, *X*_1_ = 1 and *X*_2_ = 0 for the reserve of metabolic syndrome, and *X*_1_ = 1 and *X*_2_ = 1 for metabolic syndrome; the drinking habits: *X*_1_ = 0 and *X*_2_ = 0 for “rarely drink (cannot drink)”, *X*_1_ = 1 and *X*_2_ = 0 for “sometimes” and *X*_1_ = 1 and *X*_2_ = 1 for “everyday”.

For multivariable modeling, the appropriate R packages were used as follows: LogReg was performed with glm() with a family =“binominal” argument in the “stats” package; SVM was performed with ksvm() in the “kernlab” package (kernlab v0.9–27) [24]; RF was performed with randomForest() in the “randomForest” package (randomForest, Version 4.6–14) [20]; NB was performed with NaiveBayes() in the “klaR” package (klaR, Version 0.6–14) [25].

### Variable selection

In the variable selection, graphical modeling [26] was performed so as not to select duplicate variables from the same cluster showing strong correlation by considering multicollinearity [27] with the R packages “corpcor (corpcor, Version 1.6.9)” [28] and “qgraph (qgraph, Version 1.5).” [29] The strength of correlation was calculated with a Pearson's product moment correlation coefficient. For the model performance comparison of the models, 10-fold cross-validation [30] was performed for each model.

The procedure of the 10-fold cross-validation was as follows. First, the dataset was divided into ten fractions, and the first 1/10 fraction was used as a holdout set. Model training was performed with the remaining 9/10 fractions, and then the 1/10 holdout set was used for evaluation of the trained model, from which the values of certain evaluation indices were recorded. After that, the first 1/10 holdout was returned to the original data and the next 1/10 holdout set was taken out. This training and testing of the model were performed in an iterative manner until each of the fractions had been used for as a holdout set.

### Evaluation of discrimination performance and model selection

Accuracy, error rate, sensitivity [31], specificity [31], positive predictive value (PPV) [32], negative predictive value (NPV) [32], and Area Under the ROC Curve (AUC) [33] were used for as evaluation indices. The accuracy was calculated by (number of true positive + number of true negative)/total population. The error rate was calculated by (number of false positive + number of false negative)/total population. True positive rate (TPR, also called sensitivity) was calculated by number of true positive/number of positive. True negative rate (TNR, also called specificity) was calculated by number of true negative/number of negative. Meanwhile, PPV was calculated by number of true positive/number of predicted positive and NPV was calculated by number of true negative/number of predicted negative. The ROC curves were plotted using the R packages “plotROC (plotROC, Version 2.2.1)” [34] and “ggplot2 (ggplot2 3.0.0)” [35].

The average values of the 10-fold cross-validations of each index were compared. Finally, based on the accuracy comparison among the four models, the model showing good accuracy in addition to good clinical interpretation was selected and established as the discriminant model for identifying the patients with cerebral white matter lesions.

## Results

### Assessment of each clinical examination data and questionnaire

**Fig 1:**
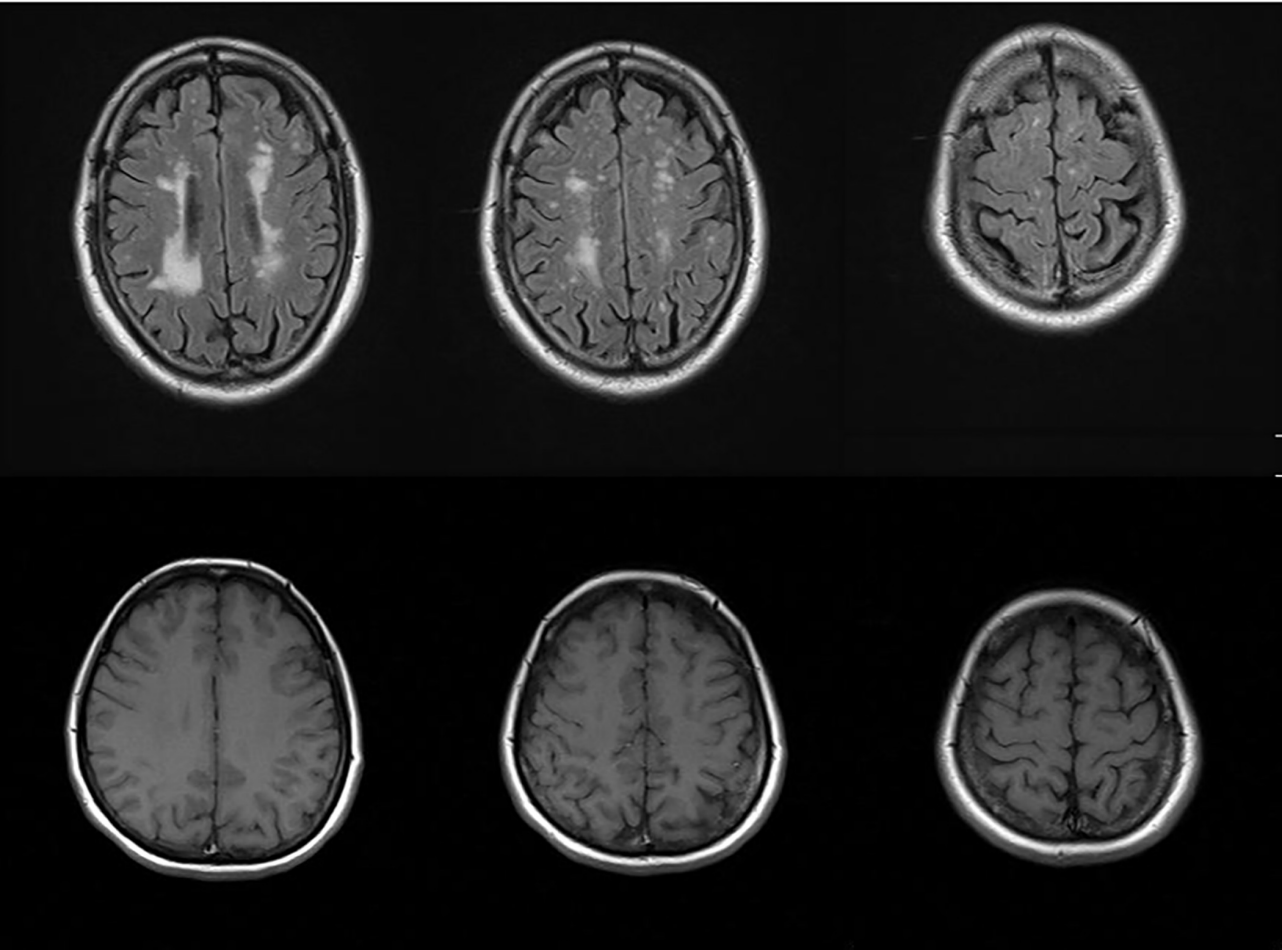
Typical examples of the presence or absence of cerebral white matter lesions. The upper three images are those of a subject with cerebral white matter lesions, while the lower three images are those of a subject without cerebral white matter lesions. T1 weighted images (T1WI), T2 weighted images (T2WI), and Fluid Attenuated Inversion Recovery (FLAIR) images were obtained using MRI equipment (MAGNETOM Symphony and MAGNETOM ESSENZA; Siemens Healthineers Global), but only the FLAIR images are shown here.

Using the head MRI, which is the gold standard for detection of white matter lesions, 1,044 out of 1,904 subjects (54.8%) were diagnosed with white matter lesions. Fig 1 shows some typical head MRI examples of the presence or absence of white matter lesions. In Fig 1, the association between the presence or absence of white matter lesions and other results from the clinical investigations, i.e., the general inspection, blood and biochemical tests, ultrasonic testing, and specific health examination questionnaire, were investigated.

Table 1 shows the assessment of each clinical examination data and questionnaire. In the general inspection, significant differences in age, gender, SBP, DBP, and the presence of metabolic syndrome were found between groups. In the blood tests, significant differences were seen in HDL, HbA1c, and BS. In the ultrasonic tests, significant differences were found in PS and n-plaque. In the answers to the specific health examination questionnaire, significant differences were seen in the patients’ experience with medications to reduce blood pressure and those to reduce blood sugar or insulin injection, as well as drinking habits, drinking volume, and smoking habits.

**Table 1:** Assessment of each clinical examination data and questionnaire.

		non white matter		white matter		
n		860		1044		
factor						*p*-value
PS, mean (sd)		0.59	(1.3)	1.49	(2.3)	<0.001
age, mean (sd)		49.96	(10.8)	61.73	(9.2)	<0.001
LDL, mean (sd)		119.64	(31.6)	121.88	(29.4)	0.109
HDL, mean (sd)		59.47	(14.7)	62.49	(15.8)	<0.001
LH, mean (sd)		2.14	(0.8)	2.08	(0.7)	0.068
TG, mean (sd)		115.59	(124.4)	108.71	(73.6)	0.134
HbA1c, mean (sd)		5.66	(0.6)	5.86	(0.7)	<0.001
BS, mean (sd)		102.41	(16.8)	105.45	(19.2)	<0.001
SBP, mean (sd)		119.96	(16.6)	127.1	(19.3)	<0.001
DBP, mean (sd)		72.43	(11.7)	75.05	(12.5)	<0.001
the number of plaque, mean (sd)		0.34	(0.7)	0.84	(1.2)	<0.001
BMI, mean (sd)		23.13	(3.4)	23.17	(3.4)	0.774
gender, n (%)	male	497	(57.8)	491	(47.0)	<0.001
	female	363	(47.2)	553	(53.0)	
metabolic syndrome, n (%)	no	687	(79.9)	742	(71.1)	<0.001
	reserve	79	(9.2)	117	(11.2)	
	yes	94	(10.9)	185	(17.7)	
smoking habit, n (%)	yes	197	(22.9)	139	(13.3)	<0.001
	no	663	(77.1)	905	(86.7)	
medication to reduce blood pressure, n (%)	yes	109	(12.7)	353	(33.8)	<0.001
	no	751	(87.3)	691	(66.2)	
medication to reduce blood sugar or insulin injection, n (%)	yes	32	(3.7)	107	(10.2)	<0.001
	no	828	(96.3)	937	(89.8)	
medication to reduce a level of cholesterol, n (%)	yes	82	(9.5)	233	(22.3)	<0.001
	no	778	(90.5)	811	(77.7)	
amount of drinking per day, n (%)	less tha 180mL	504	(58.6)	720	(69.0)	<0.001
(in terms of Sake)	180-360mL	237	(27.6)	230	(22.0)	
	360mL-540mL	89	(10.3)	69	(6.6)	
	more than 540mL	30	(3.5)	25	(2.4)	
drink habit, n (%)	rarely drink	331	(38.5)	465	(44.5)	0.028
	sometimes	269	(31.3)	296	(28.4)	
	everyday	260	(30.2)	283	(27.1)	

*p*-values were calculated by the Student's *t* test for continuous variables and by the Fisher's exact test for categorical variables. “LH” shows the LH ratio. The other abbreviations are shown in “the Materials and Methods” section.

### Variable selection for multivariate analysis

**Fig 2:**
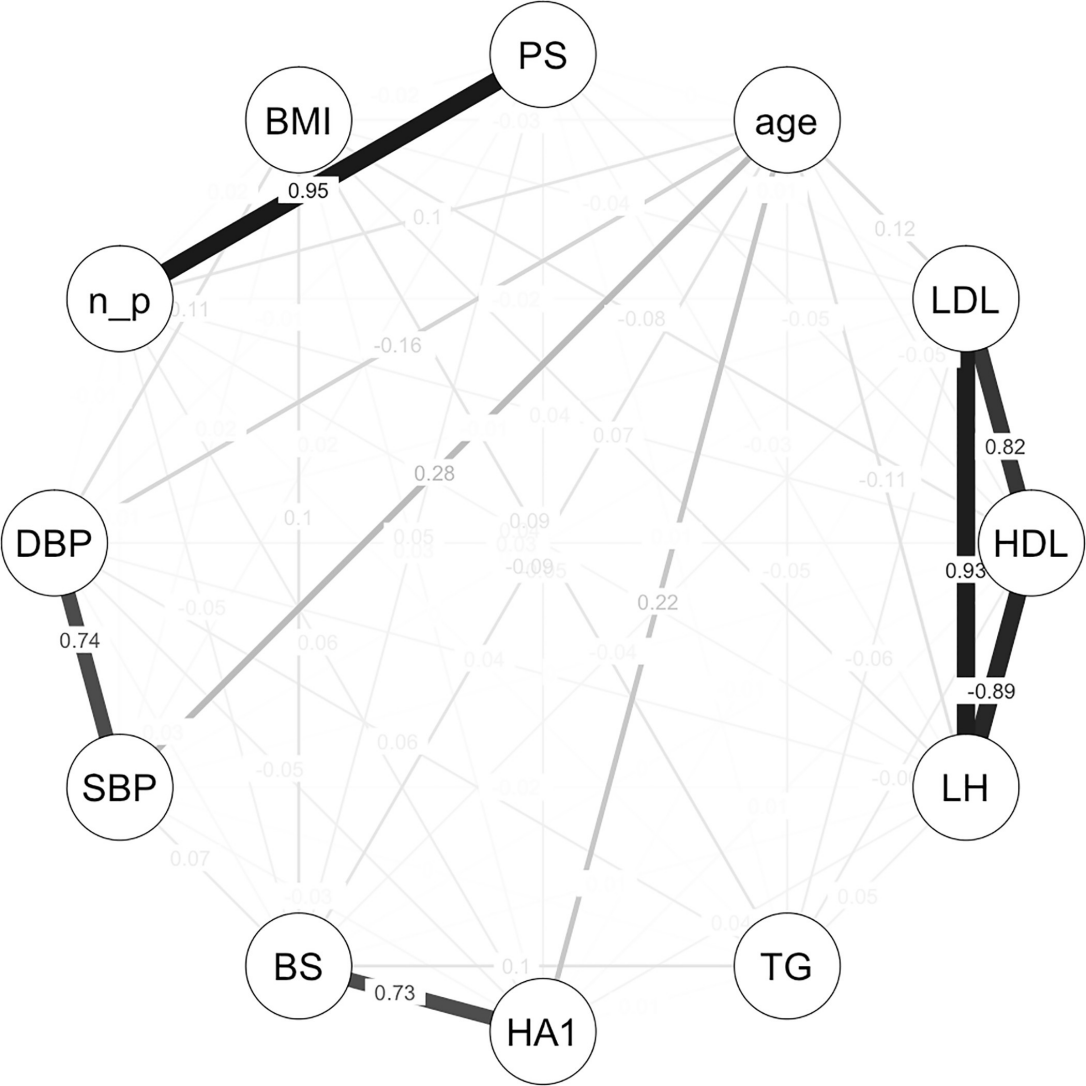
Variable selection by graphical modeling. The graphical modeling was performed using the R packages “corpcor” [26] and “qgraph” [27].

Graphical modeling was performed to avoid the multicollinearity problem that can result from the interactions among explanatory variables when creating a mathematical model. Fig 2 shows the results of the graphical modeling for variable selection. Four clusters of variables with strong correlation were created. The first cluster contained PS and n-plaque; therefore, PS was selected from this cluster because it could capture the effects of both plaque number and plaque thickness [36]. The second cluster contained SBP and DBP; SBP, which is generally considered to be a risk factor of atherosclerosis [37], was selected from this cluster. The third cluster contained HbA1c and BS; HbA1c, which is thought to reflect the blood sugar level for the most recent several months, was selected from this cluster. The fourth cluster contained LDL, HDL, and LH ratio; LDL, also called "bad" cholesterol, which is regarded to be a risk factor of arteriosclerosis [38], was selected from this cluster. From the specific health examination questionnaire, experience with medications to reduce blood pressure, experience with medications to reduce blood sugar or insulin injection, experience with medications to decrease the level of cholesterol or of neutral fat, and drinking habits [39] were selected.

### Performance comparison among mathematical models

**Fig 3:**
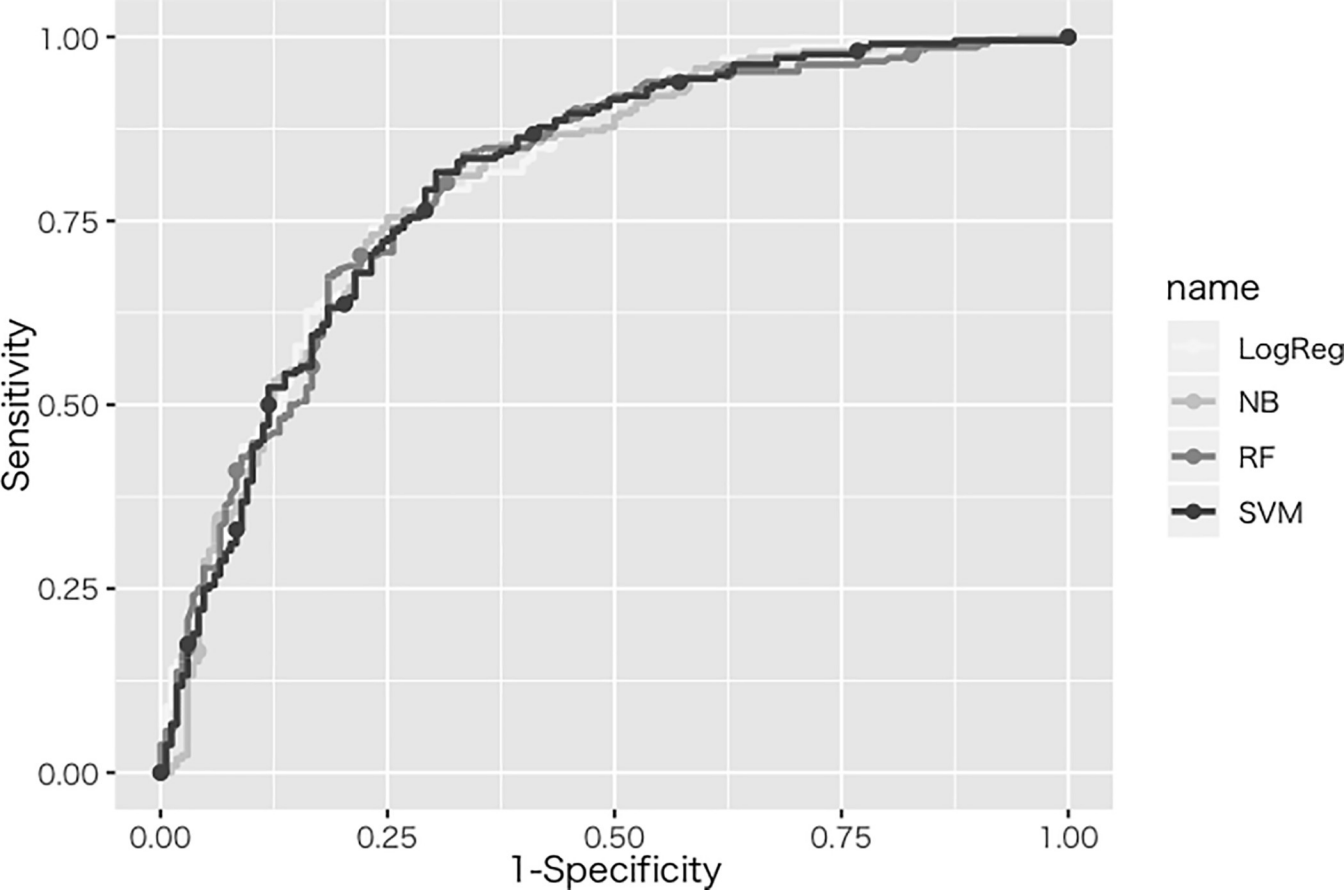
ROC curves using all the investigated models. LogReg: logistic regression analysis. NB: Naive Bayes classifier. RF: random forest. SVM: support vector machine. The ROC curves were plotted using the R packages “plotROC” [32] and “ggplot2” [33].

Table 2 shows the results of the performance comparison among models by 10-fold cross-validation. The Youden Index [22, 23] was used as the most appropriate cutoff value to evaluate the performance of each model. NB showed the highest average accuracy of 72.0% in the holdout datasets; RF showed the highest average sensitivity of 83.1%; in specificity and PPV, logistic regression analysis (LogReg) showed the highest averages, 79.4% and 79.1%, respectively; in NPV, Naive Bayes classifier (NB) showed the highest average of 69.8%; and in AUC, LogReg showed the highest average of 0.799. Fig 3 shows the ROC curves with each model using the same test data set, and they are almost overlapping with all the models.

**Table 2:** Performance comparison of each model by 10-fold cross-validation.

model	AUC	Cut off point	accuracy	error	TPR	TFR	PPV	NPV
LogReg	0.799	0.566	71.1%	28.9%	64.4%	79.4%	79.1%	64.8%
NB	0.776	0.382	72.0%	28.0%	76.5%	66.6%	73.8%	69.8%
SVM	0.787	0.679	70.7%	29.3%	64.5%	78.7%	48.8%	28.0%
RF	0.790	0.428	71.7%	28.3%	83.1%	58.0%	39.8%	30.6%

The four models, logistic regression (LogLeg), Naive Bayes classifier (NB), support vector machine (SVM), and random forest (RF), were compared with 6 indices: accuracy, error rate (error), true positive rate (TPR, also called sensitivity), true negative rate (TNR, also called specificity), positive predictive value (PPV), negative predictive value (NPV), and Area Under the ROC Curve (AUC).

### Creation of a clinical predictive model

Finally, the LogReg model was selected as a clinical predictive model because it showed the highest score in three of the six indices (specificity, PPV and AUC) by 10-fold cross-validation, and because of its clinical interpretability (e.g., odds ratio), usability, and model simplicity.

The final discriminant model created in this study is as follows:

$$\begin{array}{l} log\frac{Pr{{(Y}_{i}=1|X}_{i}=x_{i})}{Pr{{(Y}_{i}=0|X}_{i}=x_{i})}=-0.28{+1.1x}_{i1}{+0.45x}_{i2}{+0.16x}_{i3}{+0.06x}_{i4}{+0.12x}_{i5}{-0.04x}_{i6}\\ \;{+0.43x}_{i7}{+0.15x}_{i8}{+0.42x}_{i9}{+0.37x}_{i10}{+0.15x}_{i11}{+0.24x}_{i12}{+0.04x}_{i13} \end{array}$$

where the presence or absence of white matter lesions was used as the dependent variable *Y* (*Y* = 1 when white matter lesions were present, and *Y* = 0 when white matter lesions were absent). The explanatory variables were defined as follows: *X*_1_ showed age; *X*_2_ showed sex (*X*_2_ = 0 for male and *X*_2_ = 1 for female); *X*_3_ showed PS; *X*_4_ showed LDL; *X*_5_ showed SBP; *X*_6_ showed HbA1c; *X*_7_ and *X*_8_ showed the determination of metabolic syndrome (*X*_7_ = 0 and *X*_8_ = 0 for non-metabolic syndrome; *X*_7_ = 1 and *X*_8_ = 0 for the reserve of metabolic syndrome; *X*_7_ = 1 and *X*_8_ = 1 for metabolic syndrome); *X*_9_ showed the experience with medication to reduce blood pressure (*X*_9_ = 0 for “No”; *X*_9_ = 1 for “Yes”); *X*_10_ showed the experience with medication to reduce blood sugar or insulin injection (*X*_10_ = 0 for “No”; *X*_10_ = 1 for “Yes”); *X*_11_ showed the experience with medication to reduce the level of cholesterol or of neutral fat (*X*_11_ = 0 for “No”; *X*_11_ = 1 for “Yes”); *X*_12_ and *X*_13_ showed the drinking habits (*X*_12_ = 0,*X*_13_ = 0 for “rarely drink (cannot drink)”; *X*_12_ = 1, *X*_13_ = 0 for “sometimes”; *X*_12_ = 1, *X*_13_ = 1 for “everyday”). For the *i*-th patient’s data *x*_*i*_, Pr(*Y*_*i*_ = 1|*X*_*i*_ = *x*_*i*_) showed the probability that the *i*-th patient had the white matter lesions, and Pr(*Y*_*i*_ = 0|*X*_*i*_ = *x*_*i*_) showed the probability that the *i*-th patient did not have white matter lesions.

Table 3 shows the odds ratios estimated by the logistic regression model. Each variable was normalized and the estimate was adjusted to the same order. Table 4 shows the results of the logistic regression analysis performed on all of the data, instead of the train/test split dataset or cross-validation data. The confidence intervals of the odds ratio of age, gender, PS, SBP, and experience with medications to reduce blood pressure showed significance, as they were all greater than one.

**Table 3:** Odds ratio of each variable estimated by the logistic regression model.

		odds ratio	95%CI		
age		2.99	2.61	3.45	*
gender	male	reference			
	female	1.57	1.22	2.03	*
PS		1.18	1.03	1.35	*
LDL		1.06	0.95	1.19	
SBP		1.12	1.00	1.26	*
HbA1c		0.97	0.84	1.11	
metabolic syndrome	no	reference			
	reserve	1.53	1.06	2.23	*
	yes	1.16	0.81	1.67	
medication to reduce blood pressure	no	reference			
	yes	1.52	1.13	2.06	*
medication to reduce blood sugar or insulin injection	no	reference			
	yes	1.45	0.83	2.61	
medication to reduce a level of cholesterol	no	reference			
	yes	1.16	0.83	1.64	
drink habit	rarely drink	reference			
	sometimes	1.28	0.98	1.67	
	everyday	1.04	0.78	1.40	

(*) indicates that the confidence interval does not include 1.

**Table 4:** Summary of the logistic regression model using selected variables.

parameter	coefficients	std	*p*-value	
(Intercept)	-0.28	0.14	0.04	*
age	1.10	0.07	<0.001	*
Gender:female	0.45	0.13	<0.001	*
PS	0.16	0.07	0.02	*
LDL	0.06	0.06	0.30	
SBP	0.12	0.06	0.05	*
HbA1c	-0.04	0.07	0.32	
metabolic syndrome:reserve	0.43	0.19	0.03	*
metabolic syndrome:yes	0.15	0.18	0.41	
medication to reduce blood pressure:yes	0.42	0.15	0.01	*
medication to reduce blood sugar or insulin injection : yes	0.37	0.29	0.20	
medication to reduce a level of cholesterol : yes	0.15	0.17	0.38	
drink habit : sometimes	0.24	0.14	0.08	
drink habiit : everyday	0.04	0.15	0.79	

“std” represents the standard error in the output of glm().

(*) indicates a significant variable (*p*<0.05).

**Fig 4:**
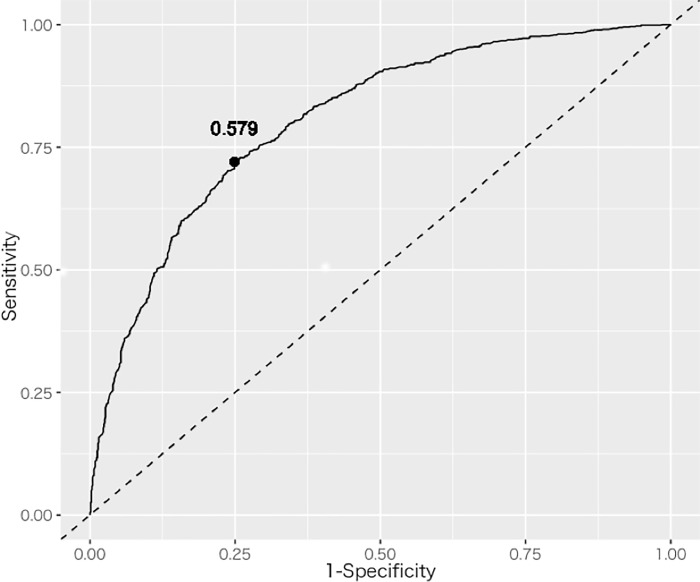
A ROC curves using the final logistic discriminant model. A point on the ROC curve shows the most appropriate cutoff value giving by the Youden Index.

The ROC curve with logistic discrimination is shown in Fig 4. In the ROC curve, for the most appropriate cutoff value, 0.579, given by the Youden Index [22, 23], the specificity was 0.249 and sensitivity was 0.720, which is the farthest point on the ROC curve from the diagonal line of AUC = 0.5.

## Discussion

In the assessment of questionnaire in Table 1, though smoking habits showed significance (*p*<0.001) in the between-groups comparison, the ratio of non-smoking patients with white matter lesions (86.7%) was higher than that of smokers with white matter lesions with smoking habits (13.3%). Generally, it is considered that smoking is a risk factor for cerebrovascular disorders [37], but this study showed the opposite result. Although it is said that smoking is a risk factor of cerebrovascular disorder [36], smoking itself is not a direct cause of ischemic change due to microangiopathy, that is, white matter lesions. It is possible that other indirect and complex factors including changes in blood constituents, such as a decrease of “good” HDL cholesterol due to smoking habits, could lead to their expression. Therefore, it is probably necessary to investigate the detailed temporal changes in smoking amounts in order to complete an effective evaluation of smoking habits. In the specific health examination questionnaire in this study, the question regarding smoking habits only asked whether the participant was a heavy smoker, defined as having smoked a total of over 100 cigarettes or have smoked over a period of 6 months, not considering the past history or cigarettes smoked or/and the number of cigarettes smoked in a day. It was considered that the index could become a more meaningful variable regarding smoking, if it included detailed information such as the smoking index represented by the product of the number of cigarettes smoked per day and the smoking history (years). However, in Japan, specific health examination questionnaires are utilized in medical checkups to check the patient's past medical history and lifestyle and to aid in patient consultation by using the similar format of questionnaires at most of the hospitals. Therefore, it is considered an important part of the patient consultation in medical checkups. Therefore, in this modeling, the specific health examination questionnaires were regarded as an important factor from the viewpoint of its practical usage.

From the assessment results of the 10-fold cross-validation, it was determined that the appropriate variable selection had been performed using the variables obtained from the limited clinical examination data so that there was not much difference in the accuracy among created models. The models used in this study include linear (LogReg) and nonlinear (SVM and RF) models as well as a stochastic model (NB). SVM [40] and RF [41] are often reported as showing better discrimination performance than linear models. Furthermore, it is considered that Bayesian modeling, which is stochastic, shows less overfitting and better generalization ability than conventional machine learning [42]. However, some contradictory reports [43–45] have appeared recently; therefore, so it was necessary to clarify the effect on the discrimination performance due to the difference in algorithms. In this study, it was demonstrated that the model algorithm did not significantly influence the discrimination performance if the appropriate variable selection had been conducted when constructing the predictive model.

Considering both the discrimination performance and the interpretability of the model, the linear model is better than the other models for the prediction of the presence of white matter lesions from clinical examination data. Using a logistic regression model, the presence of white matter lesions can be represented with a probability, and the odds ratio of a given risk factor can also be calculated. Therefore, it is considered to be suitable for clinical use among models in this study.

In this study, logistic discrimination was selected for the construction of the clinical model. There were no significant differences in accuracy, error rate, and AUC among all algorithms, but significant differences were observed in sensitivity and specificity. Generally, in screening testing before the major invasive testing, sensitivity is important in efforts to reduce the occurrence of false negatives. However, in this study, cerebral white matter lesions were predicted, a diagnosis that does not show subjective symptoms, does not lead to lethal symptoms, and does not require direct care. When diseases that cause cerebrovascular disorders are discovered, such as hypertension, hyperlipidemia, and atrial fibrillation, treatment is conducted to combat them. Considering the practical usage of the model, the model is to be used for the people getting general health examinations, by predicting the presence or absence of cerebral white matter lesions, thus allowing for the recommendation that the predicted high risk subjects receive the brain dock. Therefore, in this modeling, specificity is more important than sensitivity. By using the logistic discriminant model showing high specificity, the odds ratio of risk factors can be calculated, making the clinical evaluation seems easy.

Regarding the odds ratio of age (2.99 in Table 3), since age was standardized, the odds ratio for 1 year change in age was calculated to be 1.10, which is consistent with reports that slight white matter lesions in the elderly are an age-related phenomenon [46]. Advancing white matter lesions indicate a high risk of dementia and stroke [6], and it is important to prevent their progression.

The odds ratio of PS was significant at 1.12, which is consistent with the fact that PS is commonly used as an indicator of arteriosclerosis [47]. In the facilities investigated in this study, a PS test is performed only in the brain dock course. Given this demonstration of an association of PS with cerebral white matter lesions, if PS were to be added as an optional testing item in the comprehensive medical checkup, it would be useful as a variable for cerebral white matter lesion prediction.

Variables such as the medication to reduce blood pressure, the medication to reduce blood sugar or insulin injection, and the medication to decrease the level of cholesterol or of neutral fat are data derived from the specific health examination questionnaire and are used as a substitute for variables from the past medical history. Naturally, not all hospitals conduct a comprehensive medical checkup and there are many comprehensive medical checkups performed by private companies in Japan; therefore, it seems that there are not so many comprehensive medical checkups performed in the primary care hospitals. Therefore, because the medical history data must be obtained from the questionnaire, it is difficult to identify the exact disease name and diagnostic results without a common questionnaire format. If information can be collected in a common format, such as using hierarchical categories regarding the medical history with the questionnaire, a past history can also be used as variables, which would very likely improve the discrimination performance.

Approximately 54.8% of the patients whose data were used in this study have been diagnosed as having white matter lesions and so the prevalence is high. The brain dock course in this study is more expensive than the general comprehensive health examination courses. Consequently, it is a possibility that many examinees that had risks or medical history related to arteriosclerosis were included in the study. Because of the possibility that subjects in this study may be a high-risk group, it is not possible to conclude that the prediction of all cerebral white matter lesions can be applied for all subjects. However, it was shown to be possible to discriminate a risk group in subjects that had not received an MRI by using a prediction based on the clinical examination data.

## Conclusions

To predict cerebral white matter lesions based on clinical examination data, a logistic regression model was selected from some candidate models, created by various algorithms, based on a comparison of accuracy and interpretability. The explanatory variables of the model were age, gender, PS, LDL, SBP, and the administration of antihypertensive medication. Variable selection was important to the establishment of a high accuracy model, but the model algorithm did not significantly influence the discrimination performance if an appropriate variable selection was conducted while constructing the prediction model. This model will allow clinicians to discriminate a risk group in subjects who have not received a head MRI test.

## Supporting information

S1 Doc Questionnaire in the specific health examination, which was translated from the form in Japanese, in Shin Takeo Hospital. (DOCX)
