# Supplementary material for: Mathematical modeling for the prediction of cerebral white matter lesions based on clinical examination data
Source: PLoS One. 2019 Apr 16;14(4):e0215142. doi: 10.1371/journal.pone.0215142 (PMC6467420; doi:10.1371/journal.pone.0215142)
Supplement: S1 Doc — (DOCX) [file pone.0215142.s001.docx]

Questionnaire in the specific health examination

(in Shin Takeo Hospital: translated from the form in Japanese)

Please, select the appropriate response to the following questions.

1-3 Are you taking the following medicines at present?

1 a. Medication to reduce blood pressure

1 Yes 2 No

2 b. Medication to reduce blood sugar or insulin injection

1 Yes 2 No

3 c. Medication to reduce your level of cholesterol or of neutral fat

1 Yes 2 No

4. Have you ever been told by a doctor that you had a stroke (cerebral hemorrhage, brain infarction, etc.) and received treatment?

1 Yes 2 No

5. Have you ever been told by a doctor that you have a heart disease (angina pectoris, myocardial infarction, etc.) and received treatment?

1 Yes 2 No

6. Have you ever been diagnosed as having chronic kidney disease or kidney failure and received treatment (dialysis therapy)?

1 Yes 2 No

7. Have you ever been diagnosed as anemic?

1 Yes 2 No

8. Do you have smoking habit, or are you a heavy smoker?

“A heavy smoker” refers to those who have smoked a total of over 100 cigarettes or have smoked over a period of 6 months and have been smoking over the past month.

1 Yes 2 No

9. Have you gained over 10 kg from your weight at age 20?

1 Yes 2 No

10. Are you in a habit of doing exercise to sweat lightly for at least 30 minutes a time, 2 times weekly, for over a year?

1 Yes 2 No

11. In your daily life. do you walk or do any equivalent amount of physical activity for more than one hour a day?

1 Yes 2 No

12. Is your walking speed faster than average for those of your age and gender?

1 Yes 2 No

13. Have you gained or lost over 3 kg of bodyweight in one year?

1 Yes 2 No

14. Do you eat more quickly than other people?

1 quicker 2 normal 3 slower

15. Do you eat supper less than two hours before bedtime more than 3 times a week?

1 Yes 2 No

16. Do you eat snacks or drink sweet beverages between meals or after dinner more than 3 times a week?

1 Yes 2 No

17. Do you skip breakfast more than 3 times per week?

1 Yes 2 No

18. How often do you drink? (sake, shochu, beer, wine, whiskey, brandy, etc.)

1 everyday 2 sometimes 3 rarely drink (or cannot drink)

19. How much do you drink per day? Sake (180 ml) or a comparable quantity of other drinks, e.g. beer (500 ml), Shochu 25% (110 ml), double whiskey (60 ml), or two glasses of wine (240 ml).

1 less than 180 ml

2 180-360 ml

3 360-540 ml

4 more than 540 ml

20. Do you sleep well and enough?

1 Yes 2 No

21. Do you want to improve your life habits of eating and exercising?

1 don't want to

2 do want to

3 want to improve in near future (within a month) and began to start

4 already trying to improve (less than 6 months)

5 already trying to improve (more than 6 months)

22. Do you want to use the opportunity of health instructions for the improvement of your life habits?

1 Yes 2 No
